# Hormetic Effect of Wood Distillate on Hydroponically Grown Lettuce

**DOI:** 10.3390/plants13030447

**Published:** 2024-02-03

**Authors:** Riccardo Fedeli, Cristina Cruz, Stefano Loppi, Silvana Munzi

**Affiliations:** 1 BioAgry Lab, Department of Life Sciences, University of Siena, 53100 Siena, Italy; riccardo.fedeli@student.unisi.it; 2 Centre for Ecology, Evolution and Environmental Changes, Faculdade de Ciências, Universidade de Lisboa, 1749-016 Lisbon, Portugal; ccruz@fc.ul.pt (C.C.); ssmunzi@fc.ul.pt (S.M.); 3 BAT Center—Interuniversity Center for Studies on Bioinspired Agro-Environmental Technology, University of Naples ‘Federico II’, 80138 Napoli, Italy; 4 Centro Interuniversitário de Historia das Ciências e da Tecnologia Faculdade de Ciências, Universidade de Lisboa, 1749-016 Lisbon, Portugal

**Keywords:** antioxidants, pyroligneous acid, soilless culture, sustainable agriculture, wood vinegar

## Abstract

The addition of biostimulants to nutrient solutions of hydroponically grown crops to speed up plant growth and improve plant yield and quality has been attracting more and more attention. This study investigated the effects of wood distillate (WD) addition to hydroponically grown lettuce (*Lactuca sativa* L.) plants. Two concentrations of WD, 0.2% and 0.5%, were added to the nutrient solution, and biometric (i.e., leaf fresh weight, root fresh weight, root length and root surface area), photosynthetic (i.e., chlorophyll *a*, chlorophyll *b*, and carotenoid content) and biochemical (i.e., electrolyte leakage, total polyphenols, total flavonoids, and total antioxidant power content) parameters were evaluated. The effects of WD were hormetic, as the 0.2% concentration stimulated biometric and biochemical parameters, while the 0.5% concentration inhibited plant growth. Based on these results, it can be suggested that the addition of 0.2% WD to the nutrient solution has a stimulating effect on the growth of lettuce plants, and could be a successful strategy to boost the yield of crops grown hydroponically.

## 1. Introduction

Hydroponics has gained attention for its numerous advantages over traditional soil-based farming methods. One of the primary benefits of hydroponics lies in its precise control over the delivery of nutrients [1], which are dissolved in water and delivered directly to the plant roots. This controlled approach ensures that plants receive exactly the nutrients they require, leading to faster growth and higher yields [2] compared to their soil-grown counterparts [3,4].

A further key advantage of hydroponics is the higher water efficiency [5] compared to the conventional farming, which often requires considerable water supply. These systems are designed to be highly water efficient [5], with recirculation of water and nutrients, reducing overall consumption and waste and making hydroponics an eco-friendly solution.

Hydroponics also stands out for its space efficiency. These systems can be installed in small areas, making them ideal for urban agriculture and regions with limited land [6,7]. Vertical hydroponic systems maximize space utilization, allowing a variety of crops to be grown in defined spaces [8].

These systems are also crucial given that the estimated loss of cultivated land in Europe is approximately 280,000 ha per year [9]. This means that by 2030, the EU could lose another 4.2 million ha of cultivated land, bringing the total of abandoned areas to 5.6 million ha. Furthermore, the Food and Agriculture Organization of the United Nations (FAO) estimated that by 2050, agricultural production will have to increase by 60% to adequately meet the food needs of the world’s population, which is why the use of vertical hydroponic systems can be a very promising solution [10].

Hydroponic systems minimize the risk of pests and diseases that commonly affect soil-based plants [11], leading to healthier plants and potentially decreasing the need for pesticides, contributing to more sustainable and chemical-free agriculture. Finally, hydroponic systems allow efficient resource utilization and consistent year-round production [12,13].

To date, many different nutrient solutions have been proposed for hydroponic systems, most of them containing only inorganic ions from soluble salts of elements essential for higher plants. However, there is a growing interest towards the addition of biostimulants to the hydroponic solution, aiming at increasing crop yield and quality. Although the combination of hydroponics and biostimulants is considered as a promising eco-friendly production strategy for vegetables grown in greenhouses [14], studies regarding the direct addition of biostimulants to hydroponic solutions are still limited, and their effects on crop production still need verification.

The utilization of various natural, chemical, and physical agents to improve the quality of crops is a widely recognized and prevalent practice in the field of horticulture [15], though its impact on plants is also affected by genotype and environment [16]. Wood distillate (WD), often referred to as pyroligneous acid or wood vinegar, is one of the most interesting biostimulants available on the market. Recently, it has gained attention for its use on crops via foliar application or fertirrigation [17,18]. Wood distillate has potential applications also in hydroponics, but only few studies investigated its effects, particularly on the photosynthetic parameters of crops [19,20].

One of the primary areas of interest in the use of WD in hydroponics is its capacity to enhance nutrient uptake by plant roots [21]. This is attributed to the large presence of organic compounds within WD, such as acetic acid and various phenolic compounds [22]. These substances have been shown to potentially improve the absorption of essential nutrients by plants, leading to increased yields and quality of crops [23,24]. The precise mechanisms through which WD influences nutrient uptake are still under investigation, but early findings suggest that WD works as an eustressor, activating various defense mechanisms and physiological adaptations to cope with the stress, ultimately leading to improved resilience and growth [25]. Furthermore, WD possesses the ability to stimulate root development, induce flowering and strengthen plant resilience in the face of various environmental stressors [26,27,28]. However, it is crucial to emphasize that the effects of WD on plants are hormetic, i.e., they depend on the dosage and are also species-specific [29,30] and that excessive concentrations of WD can potentially be detrimental to plants due to its inherent acidity [31]. Consequently, it is crucial to determine the appropriate concentration of WD that maximizes its beneficial effects while avoiding any negative impact on plant growth.

Therefore, the aim of this work was to test the effects of the addition of two different concentration of WD [0.2% (*v*/*v*) and 0.5% (*v*/*v*)] to the nutrient solutions of an hydroponic system on lettuce growth by evaluating both biometric (leaf fresh weight, root fresh weight, root length, root surface area), photosynthetic (chlorophyll *a*, chlorophyll *b*, and carotenoid content), and biochemical (electrolyte leakage, total polyphenols, total flavonoids, and total antiradical activity content) parameters.

## 2. Results

The PERMANOVA analysis showed a significant effect of ‘time’, ‘treatment’, and ‘time per treatment’ on the parameters leaf fresh weight, root fresh weight, root length, root surface area, chlorophyll *a*, TPC, TFC, and ARA content. For the parameter chlorophyll *b*, the analysis showed significance of ‘time’ and ‘treatment’, while for the parameters electrolyte leakage and carotenoids it only showed significance for ‘time’ (Table 1).

### 2.1. Temporal Trends

**Figure 1:**
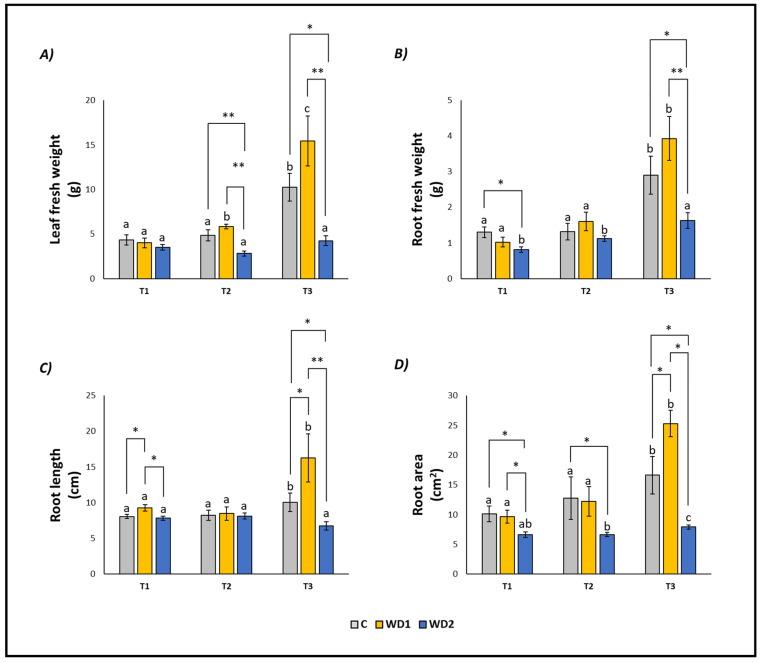
Leaf fresh weight (**A**), root fresh weight (**B**), root length (**C**), root area (**D**) of lettuce plants. C = 0% wood distillate; WD1 = 0.2% wood distillate; WD2 = 0.5% wood distillate. T1 = first harvest (three days after the start of the experiment); T2 = second harvest (nine days after the start of the experiment); T3 = third harvest (eighteen days after the start of the experiment). Different letters indicate statistically significant differences in the same treatment at different times. Asterisk (*) = indicates statistically significant differences in treatments at the same time. * = *p* < 0.05; ** = *p* < 0.01.

Leaf fresh weight, root fresh weight, root length, and root area showed similar trends (Figure 1). As expected, all the parameters in control samples (C) increased over time, following the growth of the plants, and showing significant differences at T3 (Figure 1). The same was observed in WD1 plants, with only leaf fresh weight increasing also at T2 (Figure 1). WD2 plants showed a different trend, with all parameters remaining constant (leaf fresh weight and root length) or slightly increasing at T3 (root fresh weight and root area) (Figure 1).

**Figure 2:**
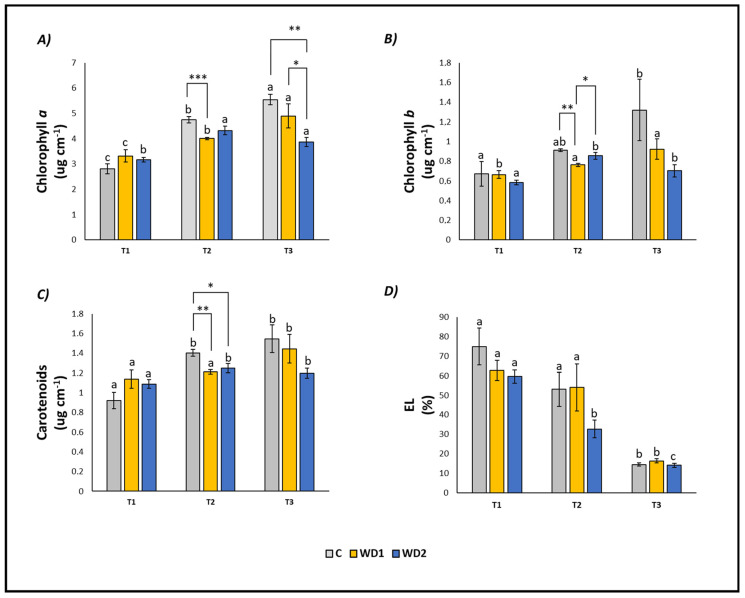
Chlorophyll *a* content (**A**), chlorophyll *b* content (**B**), carotenoid content (**C**), and electrolyte leakage (EL) (**D**) of lettuce plants. C = 0% wood distillate; WD1 = 0.2% wood distillate; WD2 = 0.5% wood distillate. T1 = first harvest (three days after the start of the experiment); T2 = second harvest (nine days after the start of the experiment); T3 = third harvest (eighteen days after the start of the experiment). Different letters indicate statistically significant differences in the same treatment at different times. Asterisk (*) = indicates statistically significant differences in treatments at the same time. * = *p* < 0.05; ** = *p* < 0.01; *** = *p* < 0.001.

The content of chlorophyll *a* (T1 < T2 < T3), chlorophyll *b* (T1 = T2 < T3) and carotenoids (T1 < T2 = T3) increased over time in C plants. Conversely, EL decreased significantly at T3 compared to T1 and T2 (T1 = T2 > T3; Figure 2). Plants treated with WD1 showed a similar trend, with a significant progressive increase in time of chlorophyll *a* (T1 < T2 < T3), chlorophyll *b* (T1 < T2 = T3) and carotenoids (T1 = T2 < T3), and a decrease in EL (T1 = T2 > T3; Figure 2). In plants treated with WD2, chlorophyll *a*, chlorophyll *b*, and carotenoids showed a significant increase at T2 and T3 compared to T1 (T1 < T2 = T3), while EL showed a significant decrease at all three investigated times (T1 > T2 > T3) (Figure 2).

**Figure 3:**
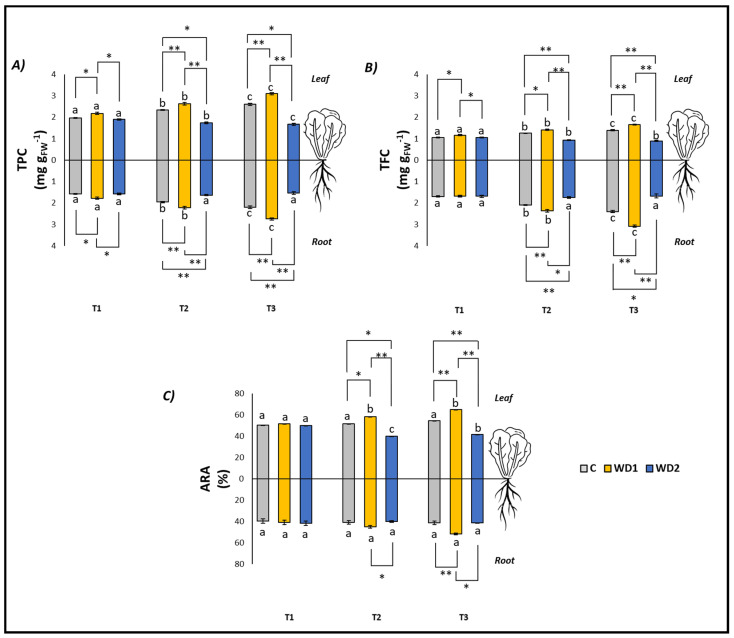
Total polyphenol content (TPC) (**A**), total flavonoid content (TFC) (**B**), antiradical activity (ARA) (**C**) of lettuce plants. C = 0% wood distillate; WD1 = 0.2% wood distillate; WD2 = 0.5% wood distillate. T1 = first harvest (three days after the start of the experiment); T2 = second harvest (nine days after the start of the experiment); T3 = third harvest (eighteen days after the start of the experiment). Different letters indicate statistically significant differences in the same treatment at different times. Asterisk (*) = indicates statistically significant differences in treatments at the same time. * = *p* < 0.05; ** = *p* < 0.01.

Regarding the antioxidant compounds (TPC and TFC, both in leaves and roots), C plants showed a significant increase at all three investigated times (T1 < T2 < T3), while no difference was found in the ARA content in both leaves and roots (T1 = T2 = T3) (Figure 3). Plants treated with WD1 also showed a significant increase in TPC and TFC (both leaves and roots) at all three investigated times (T1 < T2 < T3). ARA content in the leaves was significantly higher at T2 and T3 with respect to T1 (T1 < T2 = T3), while no differences were found in ARA content in roots over time (T1 = T2 = T3) (Figure 3). In plants treated with WD2, the leaf content of TPC and ARA showed a significant decrease in time (T1 > T2 > T3), the leaf content of TPC showed a significant progressive decrease with time (T1 < T2 < T3), and differences were found for the root content of TPC, TFC, and ARA (T1 = T2 = T3) (Figure 3).

### 2.2. Effects of WD

At T1, plants did not show any statistically significant difference in leaf fresh weight (T1 = T2 = T3); root fresh weight showed a significant difference between C and WD2 (C = WD1 < WD2); root length was significantly higher for WD1 compared to C and WD2 (C = WD2 < WD1); root area was significantly higher in C and WD1 compared to WD2 (C = WD1 < WD2; Figure 1). No significant difference was found for chlorophyll *a*, chlorophyll *b*, carotenoid, and EL (C = WD1 = WD2) (Figure 2). Regarding the antioxidant compounds, TPC (leaves and roots) and TFC (leaves) showed significantly higher values for WD1 than for C and WD2 (C = WD1 > WD2); a difference did not emerge for the content of TFC (roots) and ARA (leaves and roots) (C = WD1 = WD2) (Figure 3).

At T2, plants showed statistically significant differences in leaf fresh weight between C and WD2 and WD1 and WD2; significant differences were not found in root fresh weight, root length, and root area (C = WD1 = WD2) (Figure 1). As for the content of chlorophyll *a*, chlorophyll *b*, and carotenoids, a statistically significant difference was found between C and WD1; furthermore, for chlorophyll *b*, a statistically significant difference was found between WD1 and WD2, and for carotenoids between C and WD2; no difference was found for EL between treatments (C = WD1 = WD2) (Figure 2). Regarding the antioxidant compounds, TPC and TFC (leaves and roots) and ARA (leaves), statistically significant differences were found between all treatments with consistently higher values of WD1 (WD1 > C > WD2) (Figure 3).

At T3, plants showed statistically significant differences in leaf fresh weight and root fresh weight between C and WD2 and WD1 and WD2; root length and root area showed statistically significant differences between all treatments with higher values found in WD1 (WD1 > C > WD2) (Figure 1). Regarding the chlorophyll *a* content, statistically significant differences were found between C and WD2 and WD1 and WD2; differences were not found in the content of chlorophyll *b*, carotenoids and EL (C = WD1 = WD2; Figure 2). Regarding the antioxidant compounds, TPC and TFC (leaves and roots) and ARA (leaves) showed statistically significant differences among all treatments, with higher values found in WD1 (WD1 > C > WD2); on the other hand, ARA content in roots showed statistically significant differences between C and WD1 and WD1 and WD2 (Figure 3).

## 3. Discussion

Our findings provide insights into the dose-dependent effects of wood distillate (WD) on crops grown hydroponically. Only a few studies investigated the addition of WD directly to the nutrient solution of hydroponic systems, and the results obtained are contrasting. We observed a hormetic effect: the concentration of 0.2% WD led to a general improvement of plant growth, while at a concentration of 0.5%, WD had adverse effects on plant development. It is important to note that the biostimulant properties of WD on crops have been widely reported in the literature, typically falling within the range 0.2–0.5% for both foliar applications and fertigation [32,33]. The use of biostimulants in hydroponic systems has a boosting effect not only on plant growth, but also on crop yield and quality [34,35,36]. In our case, it is evident that WD proved to be an excellent solution for increasing the fresh weight of plants, but also the development and growth of the roots. Specifically, the root system plays a fundamental role in the growth of plants in hydroponics, which is even more important than in traditional soil growing [37]. Our results clearly show that the addition of WD to the nutrient solution showed a hormetic effect depending on the concentration used. The plants grown with WD2 showed the first negative symptoms already at T1, which became increasingly evident at T3 (18 days later). On the other hand, the results for the treatment with WD1 are completely opposed, resulting at T3 in a general improvement of all root-related parameters. Consistently with our results, Chen et al. [19] investigated the effects of WD addition in hydroponic solution on the growth of lettuce plants, founding no statistically significant difference between control plants and plants grown in the presence of 0.25% WD in terms of fresh root weight, whereas higher concentrations (0.5% and 1%) showed a statistically significant reduction in this parameter. This effect of WD on plants is commonly attributed to the different organic and inorganic components, including polyphenols, tannins, and alcohols [22]. These compounds have the remarkable capacity to stimulate plant growth by inducing a specific form of stress, known as eustress. This eustress, in turn, activates a variety of antioxidant compounds, which are crucial for the development of primary and secondary metabolic processes within the plant [38].

Antioxidant compounds play a key role in the development and overall health of plants [39]. A primary function is the protection against oxidative stress, as they neutralize harmful reactive oxygen species, safeguarding plant cells and promoting their vitality [40], also boosting the plant’s defense mechanisms. Thus, when exposed to stressors, pathogens or pests, plants often produce more antioxidants as part of their response, contributing to their overall health and resilience [40]. In addition, antioxidant compounds influence various aspects of plant growth and development by inhibiting the development of pathogens and protecting plants from disease [41]. Moreover, they are also involved in secondary metabolism, influencing the production of pigments, alkaloids and other bioactive molecules that have a major impact on plant-environment interactions [42]. They contribute to root development, promoting plant stability and aiding nutrient uptake by protecting root cells from oxidative damage [39]. The increase in the root system growth and antioxidant compound content can enhance yield and crop quality. Increased uptake of nutrients and water, as well as increased resistance to stress, can result in healthier plants [43]. Our results show that there is a positive correlation between biometric parameters and the content of antioxidant compounds (i.e., polyphenols, flavonoids, and total antioxidant power) in the plant. Indeed, the biometric parameters and antioxidant pool are statistically higher in plants treated with WD1 than in control plants, but even more so in plants treated with WD2, where the worst performance has been observed.

An increase in total polyphenol content (TPC) and total flavonoid content (TFC) has been demonstrated to be directly connected with the augmentation in antioxidant activity [44,45]. Flavonoids and polyphenols, which are often present in bio-based products, are physiologically active substances having antioxidant and anti-inflammatory properties [46]. When compared to C and WD2 plants, WD1 plants had an overall rise in both TPC and TFC in roots and leaves. To the best of our knowledge, this is the first report regarding the impact of WD on these two parameters in hydroponically grown plants. The effects of WD on antioxidant component level are limited to plants cultivated in soil. The application of WD at concentrations of 0.2% and 0.4% to tomato plants increased fruit TPC level, while only 0.4% WD increased ARA [47]; however, no increase in TPC was found in the fruits of strawberry (*Fragaria ananassa*), except for chlorogenic acid, levels of which doubled compared to the control [48].

Concerning the temporal trends observed, there were notable differences overall for all the parameters examined across the various treatments. Under normal growth conditions, plants exhibit growth over time, leading to an increase in biometric parameters (i.e., fresh leaf weight, root fresh weight, length, and area), and biochemical parameters, including the antioxidant compounds in our case. Our findings indicate that the two concentrations of WD demonstrated distinct temporal responses. Specifically, the addition of 0.2% (*v*/*v*) WD resulted in a general increase in the different parameters compared to the C plants. Conversely, the addition of 0.5% (*v*/*v*) WD led to a general reduction in the different parameters compared to both the C and WD1 plants. Therefore, it is plausible to characterize the hormetic effect of WD based on concentration, presumably attributable to the chemical composition of WD exerting a dose-dependent influence on plant growth [29].

## 4. Materials and Methods

### 4.1. Experimental Protocol and Growing Conditions

The research was carried out at the Faculdade de Ciências, of the University of Lisboa, on lettuce (*Lactuca sativa* L.), in February 2023. The experimental protocol was based on the application of the biostimulant wood distillate at two levels [0.2% (*v*/*v*) and 0.5% (*v*/*v*)] plus an untreated control (C), and the time of harvest for analytical determination (T1 = 3 days, T2 = 9 days, T3 = 18 days), using an experimental design with 10 replicates × treatment × time. Ninety lettuce seedlings were purchased from a local nursery (Horta do Campo Grande, Lisbon, Portugal). In the laboratory, after washing the roots with tap water, each seedling was placed inside a plastic basket filled with sterilized clay and placed in 15 rectangular plastic containers (22 × 8 × 33 cm) with a volumetric capacity of 8 L filled with a modified Hoagland solution (Table 2) following Cruz et al. [49]. Five of the fifteen containers were filled only with the nutrient solution, while two different treatments with wood distillate were applied in the others: WD was added to a final concentration of 0.2% (*v*/*v*) to 5 containers (WD1) and to a final concentration of 0.5% (*v*/*v*) to the remaining 5 containers (WD2). The experiment was carried out inside a climatic chamber at a temperature of 20 ± 1 °C, relative humidity (RH) of 70 ± 2% and photosynthetic photon flux density (PPFD) of 300 µmol m^−2^ s^−1^ with a 16/8 h day/night circle. At three different times (T1 = 3 days, T2 = 9 days, T3 = 18 days), ten seedlings (statistical replicates) for each treatment were removed from the hydroponic system and once brought to the laboratory were either analyzed or stored for later analyses.

#### Wood Distillate Characteristics

Wood pyrolysis results in two primary residual components: a solid fraction named biochar and WD, a liquid fraction [17]. To maximize the production of WD in comparison to biochar, the most efficient method involves fast pyrolysis carried out within the temperature range 350–500 °C. This process includes rapidly heating woody biomass followed by quick cooling. Consequently, approximately 60–75% of the resulting product constitutes the liquid phase, while the solid component comprises 15–25%, and the gaseous component contributes 10–20% to the overall product. The composition of WD primarily consists of 80–90% water, along with >300 water-soluble organic compounds, including organic acids, alkanes, phenolics, alcohols, and esters [22,50]. It is important to clarify that each WD is different being dependent on the feedstock used and the operating parameters employed during the pyrolytic process, resulting in a final product with different chemical characteristics. The WD used in this experiment (Distillato di Legno, produced by BioDea^®^ (BioDea, Arezzo, Italy) [51]) was derived from the pyrolysis of sweet chestnut (*Castanea sativa* Mill.) wood sourced from residual materials from forest management and has already been extensively studied for the cultivation of crops in soil [23,25] with important results in terms of yield and nutritional quality.

### 4.2. Plant Analysis

#### 4.2.1. Biometric Parameters

Both the leaves and the roots were initially weighed and subsequently promptly frozen at −80 °C. Photos of the roots of each sample were taken with a digital camera against a black background (Iphone 14, Apple Inc., Cupertino, CA, USA). Subsequently, the root length and the root area were assessed with the tools (i.e., Segmented line and Threeshold) available within the Fiji/ImageJ software (*v*. 1.54 h) calibrating the image scale to the relative pixels, equivalent to 1 mm.

#### 4.2.2. Pigment Content

Foliar pigments were extracted from three leaf discs (Ø 2 mm) placed in 2 mL of pure methanol kept at +4 °C for 24 h in the dark. After incubation, the absorbance of 200 μL of the sample at 470 nm, 652.4 nm, 665.2 nm, and 700 nm were measured using a UV–Vis plate reader spectrophotometer. The pigment content was calculated using the equations by Lichtenthaler and Buschmann [52]:

$$C_{a}\left(ug/cm\right)=16.72{\;A}_{665.2\;}-9.16\;A_{652.4}\;C_{b}\left(ug/cm\right)=34.09{\;A}_{652.4\;}-9.16\;A_{665.2}\;C_{\left(x+c\right)}\left(ug/cm\right)=\frac{\left(1000{\;A}_{470\;}-1.63\;C_{a\;}-104.96\;C_{b}\right)}{221}$$

#### 4.2.3. Electrolyte Leakage

Electrolyte leakage from leaf tissue was assessed using the method described by Sunkar et al. [53]. Fresh, young lettuce leaves were carefully cleansed with distilled water (dH_2_O). Subsequently, uniform pieces of leaf tissue (Ø 5 mm) were taken and soaked in 20 mL of dH_2_O at room temperature for two hours. After this soaking period, the initial electrical conductivity (EC_1_) of the solutions was recorded using a conductivity-meter. Following this measurement, the solutions were subjected to a heating process at 90 °C for 25 min. Afterwards, the solutions were allowed to cool down to room temperature before a subsequent electrical conductivity measurement (EC_2_) was carried out. The electrical leakage of the samples was quantified as a percentage, and calculated using the formula:

$$EL(\%)=(\frac{EC1}{EC2})\times 100$$

#### 4.2.4. Antioxidant Compounds

For the quantification of the total antiradical activity, 500 mg of freeze-dried lettuce leaves were subjected to homogenization in 2 mL of 80% (*v*/*v*) ethanol, followed by centrifugation at 15,000 rpm for 5 min. An aliquot of the supernatant (200 µL) was mixed with 1 mL of a solution containing 2,2-Diphenyl-1-picrylhydrazyl (DPPH), which had been previously prepared by dissolving 1.85 mg of the compound in 50 mL of 80% methanol (*v*/*v*). To facilitate a comparative analysis of the antioxidant potential, a blank and a control were prepared: the former consisting of 200 µL of 80% (*v*/*v*) ethanol in 1 mL of 80% (*v*/*v*) methanol, and the latter involving 200 µL of 80% (*v*/*v*) ethanol in 1 mL of the DPPH solution. After 1 h of dark incubation, the absorbance was read at 517 nm using a UV–Vis spectrophotometer (Agilent 8453, Santa Clara, CA, USA). Results were expressed as a percentage of antiradical activity (*ARA*%) using the formula [54]:

ARA%=100×[1−(sample absorbance/control absorbance)]

The total polyphenol content (TPC) was measured using the method proposed by Lamaro et al. [55]. Briefly, 500 mg of freeze-dried lettuce leaves were homogenized in 4 mL of 70% (*v*/*v*) acetone, followed by centrifugation at 4000 rpm for 5 min. The supernatant (0.5 mL) was mixed with 3 mL of dH_2_O, 0.125 mL of Folin-Denis’ reagent (Sigma-Aldrich, St. Louis, MO, USA), 0.750 mL of saturated Na_2_CO_3_, and finally 0.950 mL of dH_2_O. After a 30-min incubation at 37 °C, the samples were centrifuged again at 4000 rpm for 5 min, after which the supernatant was taken and finally the absorbance was measured at 765 nm using a UV–Vis spectrophotometer (Agilent 8453; Santa Clara, CA, USA). Quantification was made through referencing the absorbance of the samples to a calibration curve (ranging from 5 to 20 µg mL^−1^) made with gallic acid (Sigma-Aldrich) used as a standard. Results were expressed as milligrams of gallic acid equivalent per gram of dry weight (mg_GAE_ g^−1^ dw).

The total flavonoid content (TFC) was determined following the method proposed by Heimler et al. [56]. In brief, 500 mg of freeze-dried lettuce leaves were homogenized in 2 mL of 80% ethanol, then centrifuged at 15,000 rpm for 5 min. The resulting supernatant (300 µL) was mixed with 45 µL of a 10% AlCl_3_ solution, 300 µL of a 1M NaOH solution, and 300 µL of dH_2_O. Samples were read at 510 nm using a UV–Vis spectrophotometer (Agilent 8453). Quantification was made through a calibration curve (ranging from 5 to 200 µg mL^−1)^ made with quercetin (Sigma-Aldrich) as a standard, and the results were presented as milligrams of quercetin equivalent per gram of dry weight (mg_QE_ g^−1^ dw).

### 4.3. Statistical Analysis

To test for significant effects of WD application, we carried out Permutational Uni- and Multivariate Analyses of Variance (PERMANOVA) on the responses of time and WD treatment, respectively. Univariate analyses were based on Euclidean distance matrices, while multivariate ones were based on Bray–Curtis dissimilarity matrices, calculated on untransformed data. The following settings were used for all tests: 999 unrestricted permutations of raw data, α = 0.05. Significant terms were then investigated using posterior pairwise comparisons with the PERMANOVA t statistic and 999 permutations to test for significant differences between the treatments. All the analyses were performed using the PERMANOVA routine in the program PRIMER v.6 [57], including the add-on package PERMANOVA+ [58]. PERMANOVA correctly calculates an appropriate pseudo-F statistic for each term in the model for uni- and multivariate datasets. Moreover, the permutation approach is free from many of the assumptions of parametric statistics [57].

## 5. Conclusions

This study suggests that the effect of wood distillate in plants grown in hydroponic systems is hormetic and thus depends on the specific dosage employed. While a too high concentration can have a detrimental impact on plant growth, causing partial inhibition, a proper dose can boost root and foliar development, providing an economically and environmentally sustainable replacement of traditional fertilizers. Given the pressing global need for sustainable solutions to address the escalating human population and its impact on food production, wood distillate represents a viable option to enhance both crop yield and quality in hydroponic cultivation.

## Figures and Tables

**Table 1:** Results of the PERMANOVA analyses on leaf fresh weight, root fresh weight, root length, root area, electrolyte leakage (EL), chlorophyll *a*, chlorophyll *b*, carotenoids, leaf total polyphenol content (TPC), root TPC, leaf total flavonoid content (TFC), root TFC, leaf antiradical activity content (ARA), and root ARA.

*Source of variation*		**Leaf fresh weight**		**Root fresh weight**		**Root length**		**Root area**	
	*df*	MS	*F*	MS	*F*	MS	*F*	MS	*F*
Time	2	166.95	35.09 ***	13.51	37.91 ***	36.32	5.71 ***	252.54	16.43 ***
Treatment	2	91.32	19.19 ***	3.86	10.82 ***	55.71	8.76 ***	297.37	19.35 ***
Time × Treatment	4	38.98	8.193 ***	1.69	4.753 ***	32.26	5.08 ***	78.04	5.07 ***
Residual	36	4.75		0.35		6.36		15.37	
Total	44								
*Source of variation*		**EL**		**Chlorophyll *a***		**Chlorophyll *b***		**Carotenoids**	
	*df*	MS	*F*	MS	*F*	MS	*F*	MS	*F*
Time	2	9857	50.49 ***	34.91	77.31 ***	1.35	8.63 ***	1.50	19.99 ***
Treatment	2	584.42	2.99	2.54	5.63 ***	0.63	4.05 ***	0.07	1.05
Time × Treatment	4	237.16	1.21	3.74	8.29 ***	0.32	2.04	0.25	3.38 *
Residual	36	195.24		0.451		0.16		0.07	
Total	44								
*Source of variation*		**Leaf TPC**		**Root TPC**		**Leaf TFC**		**Root TPC**	
	*df*	MS	*F*	MS	*F*	MS	*F*	MS	*F*
Time	2	0.73	74.01 ***	0.99	84.53 ***	0.18	63.21 ***	1.83	134.4 ***
Treatment	2	2.85	288.78 ***	1.65	139.87 ***	0.77	258.71 ***	1.72	126.64 ***
Time × Treatment	4	0.45	45.75 ***	0.33	28.53 ***	0.15	49.09 ***	0.63	46.29 ***
Residual	36	0.009		0.02		0.003		0.02	
Total	44								
*Source of variation*		**Leaf ARA**		**Root ARA**					
	*df*	MS	*F*	MS	*F*				
Time	2	55.33	5.69 **	59.91	5.12 *				
Treatment	2	788.3	81.16 ***	127.7	10.91 ***				
Time × Treatment	4	169.6	17.45 ***	46.46	3.97 **				
Residual	36	9.72		11.71					
Total	44								

* *p* < 0.05; ** *p* < 0.01; *** *p* < 0.001.

**Table 2:** Chemical composition of the base nutrient solution.

	(*w*/*v*)%
KNO_3_	1.5
Ca(NO_3_)_2_ 4H_2_O	1
NH_4_H_2_PO_4_	0.5
MgSO_4_ 7H_2_O	0.25
KCl	0.5
H_3_BO_3_	0.25
MnSO_4_ H_2_O	1
ZnSO_4_ 7H_2_O	1
CuSO_4_ 5H_2_O	0.5
(NH_4_)_6_Mo_7_O_2_ 4H_2_O	0.5
FeNaEDTA	2

## Data Availability

Data are available on reasonable request from the corresponding author.
